# Efficacy of melatonin for sleep disturbance following traumatic brain injury: a randomised controlled trial

**DOI:** 10.1186/s12916-017-0995-1

**Published:** 2018-01-19

**Authors:** Natalie A. Grima, Shantha M. W. Rajaratnam, Darren Mansfield, Tracey L. Sletten, Gershon Spitz, Jennie L. Ponsford

**Affiliations:** 1 Beth Israel Deaconess Medical Center, 330 Brookline Avenue, Boston, 02215 MA USA; 2 School of Psychological Sciences, Monash University, 18 Innovation Walk, Clayton Campus, Wellington Road, Melbourne, Victoria 3800 Australia; 3 Monash Lung and Sleep, Monash Health, 246 Clayton Road, Clayton, Victoria 3800 Australia; 4 Monash-Epworth Rehabilitation Research Centre, Epworth Healthcare, 89 Bridge Road, Richmond, 3121 Victoria Australia

**Keywords:** Sleep, Insomnia, Traumatic brain injury, Acquired brain injury

## Abstract

**Background:**

The study aimed to determine the efficacy of melatonin supplementation for sleep disturbances in patients with traumatic brain injury (TBI).

**Methods:**

This is a randomised double-blind placebo-controlled two-period two-treatment (melatonin and placebo) crossover study. Outpatients were recruited from Epworth and Austin Hospitals Melbourne, Australia. They had mild to severe TBI (*n* = 33) reporting sleep disturbances post-injury (mean age 37 years, standard deviation 11 years; 67% men). They were given prolonged-release melatonin formulation (2 mg; Circadin®) and placebo capsules for 4 weeks each in a counterbalanced fashion separated by a 48-hour washout period. Treatment was taken nightly 2 hours before bedtime. Serious adverse events and side-effects were monitored.

**Results:**

Melatonin supplementation significantly reduced global Pittsburgh Sleep Quality Index scores relative to placebo, indicating improved sleep quality [melatonin 7.68 vs. placebo 9.47, original score units; difference -1.79; 95% confidence interval (CI), -2.70 to -0.88; *p* ≤ 0.0001]. Melatonin had no effect on sleep onset latency (melatonin 1.37 vs. placebo 1.42, log units; difference -0.05; 95% CI, -0.14 to 0.03; *p* = 0.23). With respect to the secondary outcomes, melatonin supplementation increased sleep efficiency on actigraphy, and vitality and mental health on the SF-36 v1 questionnaire (*p* ≤ 0.05 for each). Melatonin decreased anxiety on the Hospital Anxiety Depression Scale and fatigue on the Fatigue Severity Scale (*p* ≤ 0.05 for both), but had no significant effect on daytime sleepiness on the Epworth Sleepiness Scale (*p* = 0.15). No serious adverse events were reported.

**Conclusions:**

Melatonin supplementation over a 4-week period is effective and safe in improving subjective sleep quality as well as some aspects of objective sleep quality in patients with TBI.

**Trial registration:**

Identifier: 12611000734965; Prospectively registered on 13 July 2011.

**Electronic supplementary material:**

The online version of this article (doi:10.1186/s12916-017-0995-1) contains supplementary material, which is available to authorized users.

## Background

Sleep disturbances are commonly reported following traumatic brain injury (TBI), occurring across the spectrum of severity and persisting years following injury [1, 2]. Insomnia and hypersomnia are among the most prevalent sleep disorders in TBI patients [2, 3]. Despite a high prevalence, sleep disturbances are often overlooked in the TBI population and evidence-based treatments are lacking [3]. Consequently, untreated sleep disturbances following TBI contribute to ongoing cognitive dysfunction [4], poorer rehabilitation outcomes [5], lower productivity [6] and poorer functional status [7].

Although numerous mechanisms may contribute to sleep disturbance in TBI [1], our research demonstrates that TBI is associated with attenuated and delayed melatonin profiles [8, 9]. Specifically, we observed reduced evening [9] and overnight melatonin [8] production in TBI patients compared to age and sex matched controls, with evening melatonin production positively correlated with REM sleep [9]. As endogenous melatonin has sleep-promoting effects and is involved in the circadian control of the sleep–wake cycle, attenuated melatonin profiles may contribute to sleep-related disturbances following TBI. Thus, restoring melatonin with supplementation may help to alleviate sleep disturbances in individuals with TBI. The aim of the current study was to evaluate the effect of melatonin supplementation (2 mg/d) on sleep quality in patients with TBI reporting sleep disturbances.

## Methods

### Trial design

A randomised placebo-controlled double-blind two-period two-treatment crossover phase III clinical trial was conducted. In light of the known heterogeneity between TBI patients and sleep disturbances in this population, the use of a crossover design minimised the influence of confounding covariates, such as injury-related characteristics and factors contributing to sleep disturbances, such as mood, anxiety and pain. The study was conducted in accordance with the Declaration of Helsinki and it was approved by Monash University (CF11/1900-2011001061), Epworth HealthCare (52111) and Austin Health (H2013/04950) human research ethics committees. Participants gave informed consent before taking part. The clinical trial was prospectively registered with the Australian New Zealand Clinical Trials Registry (12611000734965) in July 2011.

The study was conducted from August 2011 until November 2016, with the first patient enrolled on 9 April 2012. The 10-week study comprised a 2-week baseline run-in period followed by two treatment periods, during which participants received either melatonin or placebo for 4 weeks, followed by a crossover with the alternative treatment for a further 4 weeks, separated by a 48-hour washout period. The washout period was chosen to minimise the carry-over of treatment [10–12], and to provide time for medication turnaround and recharging of the Actiwatch.

### Participants

People with acquired brain injury from trauma (TBI) or stroke were eligible to participate in the study. However, as only three stroke patients completed treatment, this manuscript reports only on patients with TBI. Participants were community-dwelling adults, aged 18 to 65 years, who had sustained mild to severe TBI. We recruited participants from Epworth and Austin Hospitals via referrals from physicians and allied health professionals. TBI participants had a history of blunt head trauma with loss of consciousness, and initial Glasgow Coma Scale (GCS) of 3–14 and post-traumatic amnesia (PTA). All participants had an identifiable sleep complaint corroborated by a Pittsburgh Sleep Quality Index (PSQI) global score ≥8, followed by a confirmed diagnosis of chronic insomnia according to the *International Classification of Sleep Disorders, 3rd Edition* [13]. A global PSQI of 8 has been shown to have high specificity and sensitivity to a diagnosis of insomnia in patients with TBI [14]. Participants were excluded if they self-reported sleep problems, fatigue or neurological conditions prior to TBI; were pregnant (screened with a blood test); had undertaken trans-meridian travel across more than one time zone or worked night shifts in the preceding three months; were deemed at a high risk of obstructive sleep apnoea by responses on the Berlin Questionnaire; reported consuming non-prescription sleep medication, benzodiazepines or hypnotics in the preceding 6 weeks; or used illicit or psychoactive substances in the previous 12 months. Participants’ urine was screened to rule out illicit substance use. Participants were permitted to continue with ongoing rehabilitative treatment and allowable medications (refer to Table 1), provided that the dosage did not change during the study. Participants were not permitted to commence new interventions or medications during the entirety of the trial. To ensure compliance, participants were sent daily text messages asking them to consume their medication and to fill in their sleep diary. They were also contacted weekly to monitor for adverse symptoms. If new medications were needed during the trial, the participant’s prescribing physician was contacted and treatment deferred to study completion.

**Table 1:** Baseline characteristics across treatment sequence

	Treatment sequence		
TBI characteristics	Melatonin then placebo (*n* = 18)	Placebo then melatonin (*n* = 15)	Overall (*n* = 33)
Age, mean (SD), years	35 (11)	38 (11)	37 (11)
Body mass index, mean (SD), kg/m^2^	25.5 (3.7)	26.1 (4.1)	25.7 (3.8)
Males, no. (%)	11 (61)	11 (73)	22 (67)
Paid employment, no. (%)^a^	4 (22)	3 (20)	7 (21)
Months post-injury, median (IQR Q1–Q3)	61 (28–115)	25 (10–72)	46 (13–102)
Lowest GCS, median (IQR Q1–Q3), raw value	5 (3–9)	8 (3–13)	6 (3–12)
PTA duration, median (IQR Q1–Q3), days	21 (12–45)	41 (27–60)	33 (13–47)
Mild TBI, no. (%), PTA 0 to ≤ 1 day	–	2 (13)	2 (6)
Moderate TBI, no. (%), PTA >1 to ≤ 7 days	2 (11)	1 (7)	3 (9)
Severe TBI, no. (%), PTA > 7 days	16 (89)	12 (80)	28 (85)
Patients prescribed medication, no. (%)	13 (72)	6 (40)	19 (58)
Patients using analgesics, no. (%)	7 (38)	2 (13)	9 (27)
Patients using antacids, no. (%)	1 (6)	1 (7)	2 (6)
Patients using antidepressants, no. (%)	6 (33)	2 (13)	8 (24)
Patients using antiepileptics, no. (%)	4 (22)	3 (20)	7 (21)
Patients using multivitamins, no. (%)	1 (6)	1 (7)	2 (6)
Patients using NSAIDs, no.	1 (6)	2 (13)	3 (9)
PSQI, global score	10 (3)	12 (4)	11 (3)
Sleep latency, minimum, median (IQR Q1–Q3)	25 (15–55)	23 (13–52)	24 (14–52)
Sleep efficiency, %, (IQR Q1–Q3)	76 (82–86)	81 (71–83)	81 (75–83)
ESS, score	7 (4)	9 (5)	8 (5)
HADS anxiety	8 (4)	8 (5)	8 (4)
HADS depression	11 (6)	8 (6)	10 (6)
FSS, score, median (IQR Q1–Q3)	48 (39–56)	49 (42–59)	49 (41–57)
SF-36 v1			
Physical functioning (PF), score	36 (16)	42 (13)	38 (15)
Role physical (RP), score	39 (14)	38 (12)	38 (13)
Role emotional (RE), score	38 (15)	41 (11)	39 (13)
Vitality (VT), score	39 (9)	37 (10)	38 (9)
Mental health (MH), score	40 (13)	40 (9)	40 (11)
Social functioning (SF), score	38 (12)	35 (14)	37 (13)
Bodily pain (BP), score	44 (14)	40 (12)	42 (13)
General health (GH), score	42 (11)	39 (15)	40 (13)

*ESS* Epworth Sleepiness Scale, *FSS* Fatigue severity scale, *GCS* Glasgow coma scale, *HADS* Hospital Anxiety Depression Scale, *IQR* inter-quartile range, *PSQI* Pittsburgh Sleep Quality Index, *NSAID* nonsteroidal anti-inflammatory drug, *PTA* Post-traumatic amnesia, *SF-36 v1* Short-form health survey, *TBI* traumatic brain injury

^a^Paid employment reflects part-time and full-time employment

Interested participants were screened for eligibility after providing verbal consent. Their medical history was corroborated from hospital records. Participants satisfying the inclusion criteria were referred to a sleep physician (DM) for clinical examination to rule out respiratory sleep disorders according to the American Academy of Sleep Medicine’s international classification of sleep disorders [15]. All participants were deemed in good health, established by physical examination, blood biochemistry, urine toxicology and body mass index (BMI) less than 35 kg/m^2^.

### Patient involvement

The rationale for the study was based on self-reports from people with TBI who told us that sleep was a significant problem. This study involved a community-based sample, and no patients, caregivers or laypeople were involved in developing the research question or study design. Neither patients nor laypersons assisted with the conceptualisation of the study or outcome measures, the interpretation of the results or drafting the manuscript. Outcome measures were selected based on previous studies on sleep and TBI in light of their simplicity and high sensitivity and specificity. The burden of intervention on patients was not assessed, though researchers were cognisant of minimising patient burden by limiting the number of questionnaires at follow-up appointments. All follow-up appointments were arranged at the participant’s convenience and were limited to less than 1 hour. A summary of results will be disseminated to participants by way of a newsletter, and participants and their families will be encouraged to contact us with questions. Participants will be provided with resources to assist them with obtaining melatonin supplementation.

### Intervention

Participants were randomly allocated to a 4-week melatonin or placebo treatment before crossover to the remaining treatment. Pill containers were labelled with the participant’s code and featured instructions for directions as follows: Take one capsule orally at approximately the same time every night, within 2 hours of initiating sleep. To ensure compliance, participants received text messages nightly. Melatonin treatment comprised prolonged-release melatonin formula (2 mg, Circadin®, Sigma Pharmaceuticals Australia). Placebo treatment matched the melatonin treatment for appearance, consisting of mannitol (106 mg), acacia (11 mg) and pure icing sugar (106 mg), compounded by Slade Pharmacy (Melbourne, Australia). Both treatments were encapsulated in identical two-piece gelatin capsules and dispensed in identical 30-capsule containers. This preparation was selected in light of previous studies demonstrating efficacy in individuals with insomnia [16–19], and was the only preparation available in Australia at that time. To monitor for adverse events, at the end of each period, participants were asked if they experienced any of a list of symptoms in the preceding period. If endorsed, symptoms were graded mild, moderate or severe.

### Outcome measures

Age, body mass index, current employment status, TBI injury characteristics, injury date and current medication were recorded at baseline.

#### Primary end point

The primary outcomes were sleep quality measured by PSQI global scores, and sleep onset latency measured by wrist actigraphy (Actiwatch-2, Phillips Respironics, Bend, Oregon). The PSQI has 19 items assessing subjectively perceived sleep quality in the previous month, and has been used extensively in patients with TBI. Global scores combine subdomains of sleep duration, sleep disturbance, sleep latency, sleep efficiency, daytime dysfunction, overall sleep quality and medication use. The score range is 0–21, with higher values indicating poorer sleep quality. The PSQI was completed at the end of baseline and following each treatment phase.

Throughout the study, participants completed a sleep diary retrospectively upon waking to determine time of sleep onset, offset and sleep duration. This was used to corroborate actigraphy sleep variables. To facilitate compliance, participants were sent daily text messages in the morning asking them to complete their sleep diary. Actigraphy data were visually inspected to identify discrepancies between the actigraphy data and the sleep diary. When actigraphy suggested a bedtime or wake time differed by more than 60 minutes from the diary report, the actigraphy was first adjusted by 60 minutes and then by 30-minute blocks, in combination with activity and light amplitude, as used in a previous study [20]. Actigraphy amendments were undertaken prior to unblinding and verified by a second blinded researcher.

Actigraphy sleep onset latency captures the time elapsed between the start of the rest interval relative to the sleep start time, expressed in minutes. Wrist actigraphy provides a valid measure of sleep–wake behaviour [21]. Participants were instructed to wear the Actiwatch on their non-dominant wrist each day and night, except when bathing or exercising. Actigraphy was collected in 1-minute epochs, downloaded and analysed using Actiware® software version 6.0.7 (Philips, Respironics). The sensitivity was set to medium (i.e., 40 activity counts during 1 minute was quantified as awake), as this setting is moderately correlated with polysomnography sleep variables in individuals with insomnia and has been used in the TBI population [20]. Sleep onset latency was averaged during the baseline phase and across each treatment period.

#### Secondary end points

Actigraphy sleep efficiency (i.e., total sleep time divided by total duration of the sleep episode, expressed as a percentage) was calculated nightly and averaged over each treatment period. Daytime sleepiness and subjective fatigue were assessed using the Epworth Sleepiness Scale (ESS) and Fatigue Severity Scale (FSS), respectively. The ESS was modified to assess daytime somnolence in the preceding 4 weeks. Self-reported anxiety and depressive symptomology were evaluated with the Hospital Anxiety Depression Scale (HADS). Eight facets of self-reported health-related quality of life were evaluated by the short-form health survey (SF-36 v1). All self-report questionnaires were completed after baseline and following each treatment.

#### Sample size

A repeated-measures within-between interaction compromise power analysis was conducted to determine study power (G*Power 3.0.10). Power calculations with an α = .05, two repeated measures and 0.7 correlations between repeated measures indicated that 33 participants provided 82% power to detect a small effect size (*f* = .20).

#### Randomisation and blinding

Participants were randomised to treatment order (melatonin or placebo first) following the 2-week baseline. Treatment randomisation was performed by an independent researcher. Block randomisation was implemented (block size of 4), and the six possible balanced permutations for assignment to the two conditions were assigned an integer from one to six. For each participant, treatment conditions were assigned and sealed in opaque envelopes (M for melatonin first and P for placebo first) and provided to the dispensing pharmacist. Participant codes were allocated sequentially as participants enrolled, and treatments were prepared and allocated by a pharmacist not affiliated with the study. The pharmacist preparing the treatments did not interact with the researchers or participants, and treatments were collected by the research assistants. Participants received one container at the commencement of each treatment period. Participants, physicians and study researchers were blinded to medication allocation. Unblinding of treatment occurred after the final participant evaluation. Data were coded by one researcher to indicate which treatment was received first, and those data screened for outliers and missing data. Data were analysed by a separate researcher blinded to treatment allocation.

#### Statistical analyses

Intention-to-treat analysis (ITT) was conducted. Treatment efficacy was determined by comparing primary and secondary outcome measures at the end of each treatment. All variables were inspected for normality, with transformations conducted prior to data analysis. Actigraphy sleep efficiency and FSS values were negatively skewed and corrected by applying a reflected square root transformation, with values multiplied by -1 to restore directionality. ESS and actigraphic sleep onset latency were positively skewed and the distributions were normalised by applying logarithmic transformations.

A random-effects mixed-model analysis was used to model each outcome variable as a linear function of treatment (i.e., melatonin or placebo), period (i.e., differences between period 1 and period 2 for placebo-melatonin and melatonin-placebo) and sequence (i.e., participants allocated placebo-melatonin vs. participants allocated melatonin-placebo), with participant included as a random variable. Results were considered significant if the two-tailed *p* value was <0.05. Data analysis was performed using STATA (StataCorp. 2015. *Stata Statistical Software: Release 14*. College Station, TX: Stata Corp LP).

## Results

### Participant flow

**Fig. 1:**
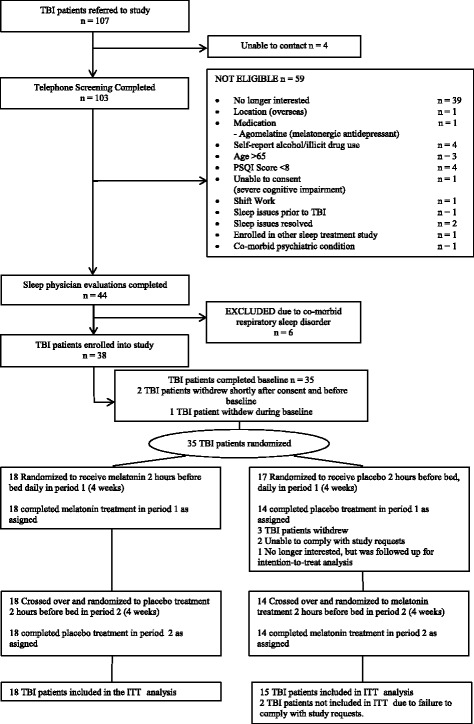
Overall patient disposition. A total of 35 participants were randomized to treatment. The final ITT sample size comprised of 33 participants. Abbreviations are as follows: *ITT* intention-to-treat, *PSQI* Pittsburgh Sleep Quality Index, *TBI* traumatic brain injury

Of the 107 TBI individuals referred to the study, 38 consented (Fig. 1) and 35 participants successfully completed baseline and were randomised to treatment. Of the 35 participants randomised, three withdrew during the placebo treatment and no participants withdrew during melatonin treatment. One withdrew after completing the questionnaires after each treatment period and was included in the ITT analysis. The final ITT sample comprised 33 TBI participants. No participant deferred treatment, with all participants receiving treatments as intended.

#### Baseline data

Sample demographics are presented in Table 1. All participants were diagnosed with chronic insomnia according to standard criteria [13]. Duration of PTA indicated most participants had severe injuries (88%). Vehicular accidents were the most frequent mechanism of TBI (*n* = 23; 70%), followed by pedestrian accidents (*n* = 6; 18%), falls (*n* = 3; 9%) and assault (*n* = 1; 3%).

#### Numbers analysed and treatment compliance

All self-report data included 33 participants, except for the HADS (*n* = 32). Actigraphic sleep latency and sleep efficiency reflected data for 31 participants. The actigraphy malfunctioned for one participant and actigraphy data were not collected from one participant who withdrew, but was included in the ITT analysis. Participants wore actigraphy on average for 25 days (Q1–Q3 = 24–28 days) when receiving melatonin and for 24 days during placebo (Q1–Q3 = 23–28 days). Paired sample *t*-tests (two-tailed) revealed no differences in the number of days of actigraphy between melatonin and placebo (*t* (31) = 1.19, *p* = .244).

The majority of participants consumed all 28 of their allocated treatments. Median treatment compliance was 100% across both treatments (melatonin Q1–Q3 = 97–100% and placebo Q1–Q3 = 96–100%). Under both conditions, participants reported taking their assigned treatment as prescribed (melatonin 118 minutes before bed, Q1–Q3 = 106–151; placebo 128 minutes before bed; Q1–Q3 = 109–170). Circadin was consumed on average at 21:16 hrs (Q1–Q3 = 21:18–21:57), while the placebo treatment was consumed at 20:45 hrs (Q1–Q3 = 20:04–21:45).

#### Primary outcomes

Melatonin was associated with a significant and moderate reduction in PSQI global scores, indicating improved sleep quality (Table 2). There was no significant reduction in sleep onset latency with melatonin compared to placebo. The sequence of treatments was not significant across primary and secondary outcome measures (data not shown). Correlational analysis did not reveal any significant association between TBI severity (as measured by PTA or GCS respectively) and effect of treatment for both PSQI or sleep onset latency (*p* > .05 for all).

**Table 2:** Mixed-model results, including mean estimate under both treatments, the difference of treatments (melatonin minus placebo), effect size and significance level

		Adjusted mean (95% CI)^a^				
	No. of participants	Melatonin treatment	Placebo treatment	Treatment effect estimate (95% CI)	Effect size^e^ (*d*)	*p* value
Primary outcomes						
PSQI, global score	33	7.68 (6.34 to 9.02)	9.47 (8.13 to 10.81)	-1.79 (-2.70 to -0.88)	0.46	<0.0001
Sleep latency, minimum^b^	31	1.37 (1.26 to 1.48)	1.42 (1.31 to 1.53)	-0.05 (-0.14 to 0.03)	0.18	0.23
Secondary outcomes						
Sleep efficiency^c^	31	-3.22 (-3.61 to -2.82)	-3.54 (-3.94 to -3.13)	0.32 (0.01 to 0.63)	0.28	0.04
ESS, score^d^	33	2.36 (2.00 to 2.73)	2.53 (2.17 to 2.90)	-0.17 (-0.40 to 0.06)	0.17	0.15
HADS anxiety, score	32	7.84 (6.23 to 9.45)	9.00 (7.39 to 10.61)	-1.15 (-1.97 to -0.34)	0.27	0.006
HADS depression, score	32	8.53 (6.93 to 10.13)	8.34 (6.75 to 9.94)	0.18 (-0.70 to 1.07)	0.04	0.68
FSS, score^c^	33	-4.18 (-4.74 to -3.62)	-3.73 (-4.28 to -3.17)	-0.45 (-0.86 to -0.04)	0.29	0.03
SF-36 v1						
Physical functioning (PF), score	33	43.17 (39.15 to 47.20)	41.72 (37.69 to 45.75)	1.45 (-0.33 to 3.24)	0.13	0.11
vRole physical (RP), score	33	37.66 (33.61 to 41.70)	38.10 (34.06 to 42.15)	-0.44 (-3.38 to 2.49)	0.03	0.77
Role-emotional (RE), score	33	37.58 (33.11 to 42.06)	36.85 (32.38 to 41.32)	0.73 (-3.38 to 4.84)	0.05	0.73
Vitality (VT), score	33	42.43 (38.97 to 45.90)	38.76 (35.30 to 42.22)	3.67 (0.36 to 6.98)	0.35	0.03
Mental health (MH), score	33	43.60 (40.00 to 47.24)	41.09 (37.45 to 44.73)	2.51 (0.58 to 4.42)	0.23	0.01
Social functioning (SF), score	33	37.09 (33.05 to 41.13)	34.82 (30.78 to 38.86)	2.27 (-1.36 to 5.90)	0.19	0.22
Bodily pain (BP), score	33	44.07 (39.85 to 48.30)	43.27 (39.05 to 47.50)	0.80 (-1.39 to 2.99)	0.06	0.48
General health (GH), score	33	40.96 (36.95 to 44.97)	40.29 (36.28 to 44.30)	0.67 (-0.97 to 2.30)	0.06	0.42

*CI* confidence interval, *ESS* Epworth Sleepiness Scale, *FSS* Fatigue severity scale, *HADS* Hospital Anxiety Depression Scale, *PSQI* Pittsburgh Sleep Quality Index

^a^Means adjusted for sequence and period

^b^Variable was log transformed

^c^Variable was reflected and square root transformed and then multiplied by -1 to restore directionality

^d^Square root transformation applied

^e^Cohens *d* effect size

#### Secondary outcomes

Relative to the placebo, melatonin was associated with a significant but small increase in actigraphic sleep efficiency, indicating a greater maximisation of sleep relative to time in bed (Table 2). Melatonin was also associated with a significant but small decrease in self-reported anxiety symptomatology, with no differences in depression. Melatonin significantly reduced the self-reported impact of fatigue during daily activities as measured by the FSS. With respect to SF-36 v1, melatonin improved self-reported vitality (VT) and mental health (MH). Melatonin was not associated with significant changes on the other six domains of the SF-36 v1, summary scores or ESS.

#### Safety

No serious adverse events were reported. Symptoms were more frequently reported during placebo treatment (Additional file 1: Table S1). The most commonly reported symptoms were neurological, followed by bodily pain, gastrointestinal and dermatologic.

#### Perceived treatment order

Participants were asked to guess treatment allocation during the preceding period. The number of participants who guessed correctly was five (28%) in the melatonin group and eight (47%) in the placebo group during period 1, and seven (41%) in the melatonin group and five (28%) in the placebo group during period 2.

## Discussion

The aim of the current study was to investigate the efficacy of melatonin in alleviating sleep disturbance in patients with TBI and insomnia. We found that melatonin improved subjective sleep quality and actigraphic sleep efficiency. Melatonin also reduced self-reported anxiety symptomatology and fatigue, whilst improving self-perceived vitality and mental functioning as assessed by the SF-36 v1 health survey. No improvement was observed for daytime sleepiness, depressive symptomatology or the six remaining SF-36 v1 domains. The present results, therefore, suggest that melatonin may be useful in treating sleep disturbances in patients with TBI.

Our study is limited by the following factors. Firstly, the sample recruited was smaller than intended. Due to the poor recruitment of stroke patients, we cannot determine whether melatonin can improve sleep following a stroke. Secondly, we did not determine endogenous melatonin concentrations and circadian phase in all participants. In a subset of TBI participants (*n* = 9), we demonstrated a 42% reduction in overnight salivary melatonin production (*d* = 0.87; *p* = .034) and delayed circadian phase (*d* = 1.23; *p* = .003) relative to age and-gender matched controls with similar sleep schedules [8]. As melatonin concentrations and phase were not captured, we are unable to determine the extent to which changes in sleep quality were mitigated by alterations in circadian phase. The aim of the present study was to demonstrate the efficacy of melatonin supplementation in TBI regardless of melatonin production and circadian phase, given that such information is not measured in the clinical setting. The current study was designed to exploit the sleep-promoting effects of melatonin, which have been previously characterised [22], utilising a prolonged-release preparation that mimics the endogenous profile and targets sleep maintenance problems. Therefore, timing of treatment was selected to harness the sleep prompting effects, with time of administration consistent with directions for use as indicated by the manufacturer. Although additional information regarding endogenous melatonin profiles would have been useful, melatonin supplementation was found to be therapeutic. Phenotyping circadian phase (e.g., dim light melatonin onset (DLMO)) would be an important step for future studies by informing the treatment approach by targeting underlying circadian misalignment for maximal benefit. Furthermore, recent discussions regarding the therapeutic outcomes between short-acting vs. long-acting melatonin preparations [23] suggest comparative studies are needed for evidence-based recommendations.

We implemented a 48-hour washout period between consecutive treatments. Although the terminal half-life for melatonin is relatively short (3.5–4 hours) [24], it is possible that melatonin administered in the first treatment period could have induced a circadian phase advance, which could have persisted for up to several days in the following (placebo) treatment period [10–12]. We confirmed that there were no treatment order effects across all outcome measures, and we limited most of the assessment treatment outcomes (except sleep diary and actigraphy) to the end of each treatment condition, when circadian phase would have re-established after melatonin treatment had ceased.

The rationale of using a crossover design over a parallel design was to minimise confounding covariates such as TBI characteristics and factors underpinning sleep disturbance, such as depression, anxiety and pain, which were inherently controlled by each participant serving as their own control. The implementation of a crossover design reduced the number of participants required, and it guaranteed all participants received the active treatment.

To our knowledge, only one other study has examined the therapeutic benefit of melatonin on sleep disturbance in TBI. Kemp and colleagues did not find any statistically significant benefits of melatonin on sleep latency, duration, quality or daytime alertness [25]. However, this study was limited to seven males and melatonin was compared to amitriptyline rather than a placebo. With a rigorous study design involving a placebo control and larger sample size, we found melatonin to be efficacious in improving sleep relative to a non-active control.

Previous clinical trials have shown melatonin’s sleep-promoting properties in patients with insomnia [16–19] and tetraplegia [26]. Trials that have utilised Circadin (2 mg), the melatonin formulation used in the current study, have shown that this melatonin preparation improves sleep quality [17–19] and sleep efficiency, [16, 27] in people over the age of 55 with insomnia. A similar melatonin formulation has been shown to be effective in improving sleep quality in patients with tetraplegia [26, 28] for whom melatonin profiles are abolished [29, 30]. Our study extends these findings by showing that a prolonged-release melatonin formation, Circadin, is efficacious in improving sleep quality and sleep efficiency in patients with TBI.

The current study found that melatonin improved subjective sleep quality. Such findings may be attributed to the sleep-promoting properties of melatonin [22]. The temporal association between the nocturnal rise in melatonin and increase in sleep propensity suggests that onset of nocturnal melatonin secretion facilitates the transition from wake to sleep by inhibiting the wake-alerting system [31, 32]. Melatonin may act as a sleep-promoting agent by attenuating wake-promoting signals from the suprachiasmatic nucleus, primarily via the melatonin MT1 receptor [33]. Melatonin’s sleep-promoting properties may also be mediated by the hypothermic response based on the temporal and causal relationship between increases in endogenous melatonin concentrations and subsequent decline in core body temperature [34, 35]. It has also been hypothesised that melatonin may act as a muscle relaxant, [36] and this may explain the apparent impact of melatonin on anxiety symptoms in the present study. The anxiolytic properties of melatonin have been demonstrated in rodents [37, 38] and paediatric populations [39]. The improvement in sleep quality also appeared to have a flow-on effect to reduce the subjective impact of fatigue in daily life and improve vitality. Although no clinical global impression was obtained in the current study, a large majority of the outcome measures were based on self-reports by the participants and thus, the current findings are clinically meaningful because changes were perceived by the participants themselves.

The present study required participants to consume treatment approximately 2 hours prior to habitual bedtime and after their evening meal. These instructions are in line with previous studies utilising Circadin [16–18, 40] and are based on the pharmacokinetic profile of Circadin (*T*_max_ = 1.6 hours) [41]. As a large majority of participants consumed their treatment as prescribed, the translation of the current findings into clinical practice is dependent on appropriately timed melatonin administration.

Various pharmacological treatments exist for the management of sleep disturbance. Hypnotics such as benzodiazepines and non-benzodiazepines are frequently prescribed to patients with TBI experiencing sleep disturbance [42]. Whereas benzodiazepines and non-benzodiazepines are equally effective in treating insomnia in patients with TBI over 7 days [43], the efficacy and safety of long-term use in patients with TBI is unknown. Hypnotics are only indicated for short-term use and prolonged use can result in dependence, especially in vulnerable populations, such as patients with TBI [44]. Conversely, the safety and tolerance profile of melatonin observed in the current study is consistent with previous studies in non-TBI populations [17]. Thus, based on the current findings, melatonin affords TBI patients an alternative treatment to alleviate sleep disturbance with minimal side effects.

In the current study, melatonin had a moderate effect size for improving sleep quality (*d =* 0.46). This magnitude of improvement in sleep quality is double that of amitriptyline (a tricyclic antidepressant) relative to no treatment, when administered to patients with TBI over the same time period (*d* = 0.21) [25]. As melatonin was well tolerated in the current study, with no serious adverse events reported, melatonin offers clinicians an alternative treatment to that of conventional sleeping medications.

While melatonin was found to be effective in alleviating sleep disturbances, it is acknowledged that the mechanisms underpinning sleep disturbances in TBI involve multiple biological and psychological systems, such as alterations in monoaminergic neurons [45, 46] and wake-promoting hypocretin-1 neurons [47], and alterations in melatonin levels [8, 9], as well as pain [48, 49] and mood disturbance [50]. This suggests that while melatonin supplementation may alleviate sleep disturbance in individuals with TBI, melatonin is unlikely to address all sleep problems. Cognitive behavioural therapy for insomnia (CBT-I) has been shown efficacious in TBI populations [51]. Due to the multi-factorial nature of sleep disturbances following TBI, complementing melatonin supplementation with CBT-I may prove beneficial.

## Conclusions

This study provides preliminary evidence for the efficacy of melatonin in alleviating sleep dysfunction in patients with TBI and insomnia. Although melatonin was well tolerated and provides a safe alternative to traditional hypnotic medications in patients with TBI and insomnia, treatment efficacy is unknown over longer durations. Future research should investigate the role of combining melatonin supplementation with other treatments, such as CBT-I and light therapy, to determine improvements in sleep outcomes in TBI beyond that of melatonin alone.

## Additional file

Additional file 1: Table S1. Frequency of symptoms for placebo and melatonin treatments. (DOCX 16 kb)

## Abbreviations

CI: Confidence interval
ESS: Epworth Sleepiness Scale
FSS: Fatigue Severity Scale
GCS: Glasgow Coma Scale
HADS: Hospital Anxiety Depression Scale
IQR: Inter-quartile range
ITT: Intention-to-treat
NSAID: Nonsteroidal anti-inflammatory drug
PSQI: Pittsburgh Sleep Quality Index
PTA: Post-traumatic amnesia
SF-36 v1: Short-form health survey
TBI: Traumatic brain injury
