# Supplementary material for: Efficacy of melatonin for sleep disturbance following traumatic brain injury: a randomised controlled trial
Source: BMC Med. 2018 Jan 19;16:8. doi: 10.1186/s12916-017-0995-1 (PMC5774131; doi:10.1186/s12916-017-0995-1)
Supplement: Additional file 1: Table S1. — Frequency of symptoms for placebo and melatonin treatments. (DOCX 16 kb) [file 12916_2017_995_MOESM1_ESM.docx]

**Supplementary Online Content**

**Grima NA, Rajaratnam MW, Mansfield, D., et al., Efficacy and safety of melatonin for sleep disturbance following traumatic brain injury: A randomised controlled trial**

**Table S1.** Frequency of Symptoms for Placebo and Melatonin Treatments

**Table S1.** Frequency of Symptoms for Placebo and Melatonin Treatments

| **Symptom category** | **Placebo Treatment** | | | | | **Melatonin Treatment** | | | | |  |
| --- | --- | --- | --- | --- | --- | --- | --- | --- | --- | --- | --- |
|  | No. ppl (%) | | Perceived severity | | | No. ppl (%) Perceived severity | | | |  | |
|  |  | Mild | | Moderate | Severe |  | Mild | Moderate | Severe | |  |
| **Dermatologic** | 6 (17) |  | |  |  | 6 (17) |  |  |  | |  |
| Body rash |  | 1 | | 2 | 1 |  | 2 | 0 | 0 | |  |
| Dry skin |  | 2 | | 2 | 1 |  | 4 | 0 | 0 | |  |
| Itchy skin |  | 1 | | 2 | 0 |  | 5 | 0 | 0 | |  |
| **Gastrointestinal** | 11 (31) |  | |  |  | 12 (34) |  |  |  | |  |
| Abdominal pain |  | 4 | | 2 | 0 |  | 2 | 1 | 1 | |  |
| Change in Appetite |  | 4 | | 0 | 1 |  | 3 | 2 | 0 | |  |
| Constipation |  | 2 | | 1 | 0 |  | 0 | 1 | 0 | |  |
| Diarrhoea |  | 2 | | 2 | 0 |  | 0 | 1 | 0 | |  |
| Flatulence |  | 4 | | 0 | 0 |  | 2 | 2 | 0 | |  |
| Nausea |  | 3 | | 2 | 0 |  | 1 | 1 | 1 | |  |
| Unusual bowl sounds |  | 2 | | 0 | 0 |  | 1 | 1 | 0 | |  |
| Emesis |  | 1 | | 1 | 0 |  | 0 | 1 | 0 | |  |
| **Bodily Pain** | 14 (40) |  | |  |  | 10 (29) |  |  |  | |  |
| Neck |  | 2 | | 5 | 2 |  | 4 | 2 | 2 | |  |
| Back |  | 1 | | 8 | 2 |  | 2 | 1 | 3 | |  |
| Joint |  | 5 | | 5 | 0 |  | 0 | 2 | 2 | |  |
| **Neurologic** | 18 (51) |  | |  |  | 15 (43) |  |  |  | |  |
| Blurred vision |  | 2 | | 3 | 1 |  | 2 | 1 | 2 | |  |
| Dizziness |  | 7 | | 2 | 0 |  | 5 | 4 | 1 | |  |
| Drowsiness |  | 6 | | 4 | 2 |  | 3 | 1 | 3 | |  |
| Migraine |  | 2 | | 5 | 1 |  | 6 | 3 | 1 | |  |
| Vivid dreams |  | 1 | | 1 | 1 |  | 2 | 1 | 0 | |  |
